# SURROGATES’ DECISION-MAKING CONFIDENCE AND COLLABORATIVE WILLINGNESS ON THE POST: THE ROLE OF SOCIAL NORMS

**DOI:** 10.1093/geroni/igz038.502

**Published:** 2019-11-08

**Authors:** Rachael Spalding, Jenna Wilson, Barry Edelstein

**Affiliations:** West Virginia University, Morgantown, West Virginia, United States

## Abstract

When patients become incapacitated due to illness or frailty, “surrogates” work with patients’ providers to make medical decisions on their behalf. In surrogate decision-making situations, surrogates’ decision-making confidence predicts collaborative willingness, or the extent to which they are willing to work with the patient’s providers when making decisions (Spalding & Edelstein, under review). In an attempt to explain this finding, the current study examined whether perceived social norms for patient-physician collaboration and another psychological variable, consideration of future consequences, mediated the relation between decision-making confidence and collaborative willingness. Participants (n= 172) from Amazon’s Mechanical Turk completed self-report measures and a hypothetical surrogate decision-making task. A parallel multiple mediation analysis using 5000 bootstrapped samples with the PROCESS macro (Hayes, 2013) was conducted. The overall model explained 43.4% of the variance in collaborative willingness, F(4, 166) = 9.59, p< .001. There was a significant indirect effect of decision-making confidence on collaborative willingness through perceived social norms (b= .068, SE= .034, 95% CI [.014, .154]). There was not a significant indirect effect through consideration of future consequences. After including the significant indirect path through perceived social norms, the direct effect (b= .348, p< .001) of decision-making confidence on collaborative willingness was reduced (b= .243, p= .003). Thus, perceptions of social norms partially accounted for the relation between decision-making confidence and collaborative willingness. This finding illustrates how social perceptions of patient-provider collaboration can facilitate desirable medical decision-making behaviors, such as collaboration.

